# Strategies for Improving Bioavailability, Bioactivity, and Physical-Chemical Behavior of Curcumin

**DOI:** 10.3390/molecules27206854

**Published:** 2022-10-13

**Authors:** Levente Zsolt Racz, Csaba Pal Racz, Lucian-Cristian Pop, Gheorghe Tomoaia, Aurora Mocanu, Ioana Barbu, Melinda Sárközi, Ioana Roman, Alexandra Avram, Maria Tomoaia-Cotisel, Vlad-Alexandru Toma

**Affiliations:** 1 Research Center in Physical Chemistry, Faculty of Chemistry and Chemical Engineering, Babes-Bolyai University of Cluj-Napoca, 11 Arany Janos Str., RO-400028 Cluj-Napoca, Romania; 2 Department of Orthopedics and Traumatology, Iuliu Hatieganu University of Medicine and Pharmacy, 47 Gen. Traian Mosoiu Str., RO-400132 Cluj-Napoca, Romania; 3 Academy of Romanian Scientists, 3 Ilfov Str., RO-050044 Bucharest, Romania; 4 Faculty of Biology and Geology, Babes-Bolyai University, 4-6 Clinicilor Str., RO-400006 Cluj-Napoca, Romania; 5 SC. Parapharm SRL, 9 Dacilor Str., RO-335200 Brad, Romania; 6 Institute of Biological Research, Branch of NIRDBS Bucharest, 48 Republicii Str., RO-400015 Cluj-Napoca, Romania

**Keywords:** bioavailability, therapeutic properties, protein–curcumin interactions, whey proteins, β-lactoglobulin, curcumin complexes

## Abstract

Curcumin (CCM) is one of the most frequently explored plant compounds with various biological actions such as antibacterial, antiviral, antifungal, antineoplastic, and antioxidant/anti-inflammatory properties. The laboratory data and clinical trials have demonstrated that the bioavailability and bioactivity of curcumin are influenced by the feature of the curcumin molecular complex types. Curcumin has a high capacity to form molecular complexes with proteins (such as whey proteins, bovine serum albumin, β-lactoglobulin), carbohydrates, lipids, and natural compounds (e.g., resveratrol, piperine, quercetin). These complexes increase the bioactivity and bioavailability of curcumin. The current review provides these derivatization strategies for curcumin in terms of biological and physico-chemical aspects with a strong focus on different type of proteins, characterization methods, and thermodynamic features of protein–curcumin complexes, and with the aim of evaluating the best performances. The current literature review offers, taking into consideration various biological effects of the CCM, a whole approach for CCM-biomolecules interactions such as CCM-proteins, CCM-nanomaterials, and CCM-natural compounds regarding molecular strategies to improve the bioactivity as well as the bioavailability of curcumin in biological systems.

## 1. Introduction

Medicinal herbs have been used for the treatment of many diseases since the beginning of civilization. A significant part of the world’s population living in developing countries believes in traditional herbal medicines for their primary health care [1]. Even in the modern allopathic system, medicinal plants play a key role in public health care [2]. The products of natural origin and the molecules extracted from them have represented since ancient times an important raw material for medicine and the discovery of new drugs [3]. A plant that has been widely studied is *Curcuma longa* L. [4] or turmeric, which has attracted the interest of researchers and been the focus of numerous scientific studies. Turmeric is a common food spice, used as a traditional medicine in India and China to treat wounds, skin diseases, eye infections, dental, and digestive disorders [5,6].

**Figure 1:**
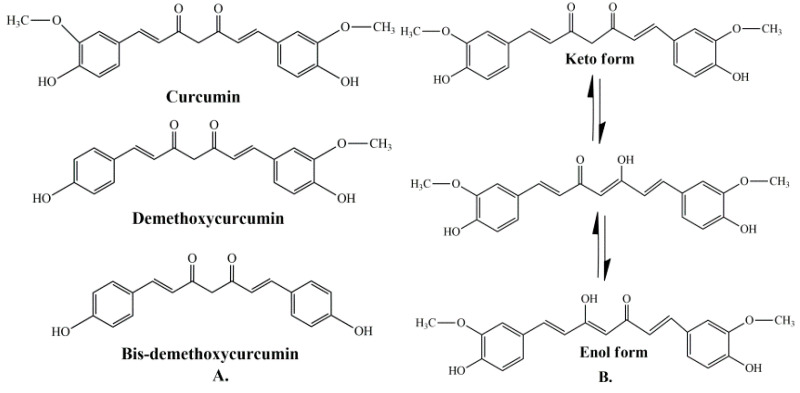
(**A**) Structure of curcumin (CCM), demethoxycurcumin (DMC), and bis-demethoxycurcumin (BMC) [20]. (**B**) The enol and keto forms of CCM [20].

Curcumin represents a naturally occurring polyphenolic component, isolated from the rhizomes of *Curcuma longa* L. Curcuminoids are natural chemical compounds found in turmeric, which are responsible for its orange color. Turmeric rhizomes contain 3–5% of curcuminoids derivatives [7], including demethoxycurcumin (10–20%), bis-demethoxycurcumin (3%), and curcumin (CCM) (75–85%), which is the most important bioactive constituent [8,9]. All three curcuminoids derivatives have antioxidant abilities and antifungal activity [10]. The highest values of these properties are in the case of CCM, followed by demethoxycurcumin (DMC) and bis-demethoxycurcumin (BMC) (Figure 1A) [11]. Many studies have indicated that demethoxycurcumin and bis-demethoxycurcumin have therapeutic uses for anti-Alzheimer’s [12] anti-cancer [13] and anti-inflammatory treatment [14,15]. Huang et al. (2020) have shown that CCM, DMC, and BMC decreased the viability of cancer cells, but their synergistic use is more effective in the treatment of osteosarcoma [16].

Curcuminoids are the major component of turmeric, which are responsible for its orange color.

The structure of curcumin includes the central 1,6-heptadiene-3, and 5-dione linked with 2 terminal phenolic rings (Figure 1B). The presence of intramolecular hydrogen atoms transferred at the β-diketone chain of CCM determines the keto-enol tautomeric conformations in equilibrium [17]. Under low pH conditions (pH 3–7), the CCM molecules exist in di-keto form, where CCM behaves as a strong proton donor. However, in alkaline conditions (pH > 8), the enolate form prevails, and CCM behaves as an electron donor. The existence of an enolate form in solution has been proven to be central to the radical scavenging capability of CCM [18,19].

Taken together, the biological and chemical features of curcumin exposed in the current context highlight the need to approach from a new thermodynamic perspective as well as bioavailability with respect to the pharmacological or biotechnological uses of curcumin. In the current review, the objective is outlined around a series of molecular strategies regarding the optimization of curcumin bioavailability in various biological systems. Furthermore, a central part of this review is dedicated to methodologies used to enhance the curcumin bioactivity and bioavailability focusing on the formation of complexes or on the self-assemblies made of curcumin with proteins or with other bioactive polymers, such as polyethylene glycol, or other biological molecules, such as piperine or resveratrol. In addition, we summarize and discuss various methods used to characterize the formed complexes or used for monitoring the interaction in these complex systems.

## 2. Biological Actions of Curcumin

### 2.1. General Bioactivities and Limitations

**Figure 2:**
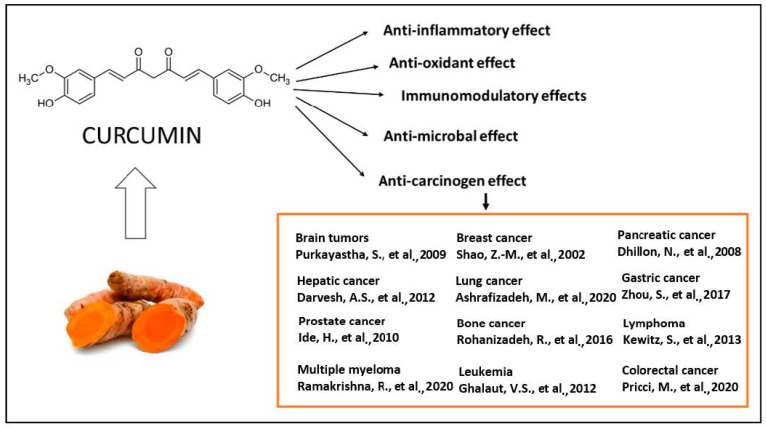
The therapeutical effects of CCM are reflected by a large spectrum of action, as reported by the literature. The antitumor action of CCM was extensively explored in clinical and experimental conditions [22,23,24,25,26,27,28,29,30,31,32,33] as well as the antioxidant [34], anti-inflammatory [35,36,37], antibacterial [38,39,40] and immunomodulatory effects [41].

The majority of systemically administered synthetic drugs has undesirable side effects or toxicity and can spread to nontarget body parts. For this reason, it is desirable to use safe compounds that also present a good bioavailability. CCM is a hydrophobic polyphenol [21], with numerous pharmacological properties (Figure 2), such as antiproliferative, anticancer [22,23,24,25,26,27,28,29,30,31,32,33], antidiabetic, antioxidant [34], and anti-inflammatory activities [35,36,37] and antimicrobial properties [38]. In general, the well-documented antiviral [38,39,40], anti-inflammatory, and immunomodulatory effects [41] of CCM, along with the evidence on anti-fibrotic [42] and lung protective properties [43] of this phytochemical in lung tissue, make it a likely molecule for the prevention and treatment of the infection with the SARS-CoV19 virus [44]. The virus binds to the target cell receptor, the angiotensin converting enzyme 2 receptor (ACE2R). Research has proved that CCM inhibits this virus ACE2R by inhibiting the spike protein and the function of the ACE2 receptor [45].

CCM is a unique molecule due to its wide range of useful biological functions. Clinical studies have shown that a daily dose of 8 g (considered high) of CCM has no adverse effects on the human organism [46].

**Figure 3:**
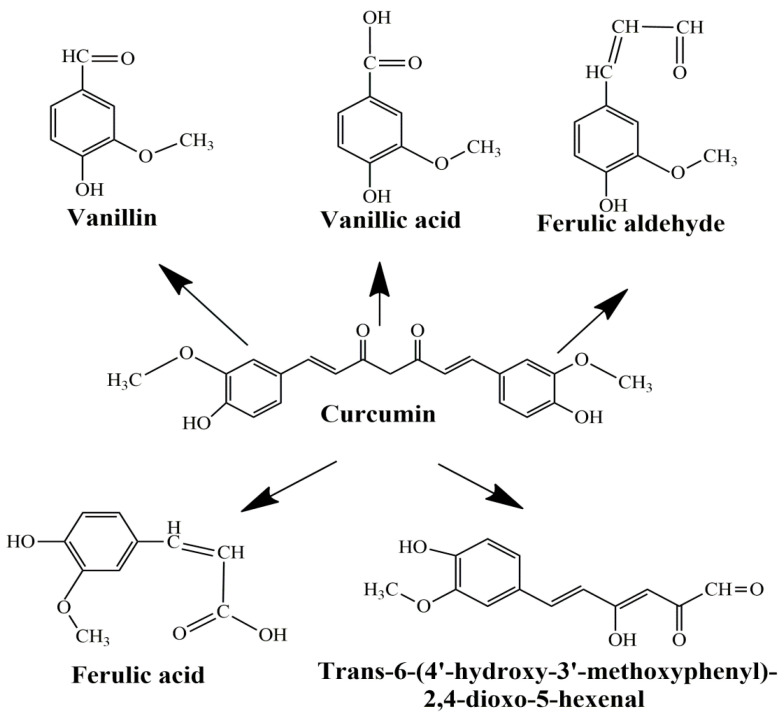
Product decomposition of curcumin in alkaline condition [56,57].

The main limitations of CCM are low water solubility, low absorption, rapid systemic elimination from the human body, degradation at alkaline pH, and limited oral bioavailability [47,48,49]. Under alkaline conditions, CCM degrades to *trans*-6-(40-hydroxy-30-methoxyphenyl)-2, 4-dioxo-5-hexanal, ferulic acid, feruloyl methane, and vanillin (Figure 3) very quickly (~30 min) [50,51], while under acidic conditions, less than 20% of CCM was decomposed at 1 h. Only a small portion of CCM ingested orally is adsorbed in the intestine because a major part of CCM is excreted [49]. In addition, the absorbed CCM went through a quick metabolism in the liver and plasma [52]. Several studies show that decomposition products such as ferulic acid and vanillin also have antimicrobial, antiarrhythmic, antithrombotic, and antioxidant properties and can inhibit cancer development [53,54]. However, the biological activities of ferulic acid and vanillin are weaker compared to CCM [55].

### 2.2. Antibacterial Properties

Although progress has been made over the years in the development of methods to treat infections caused by bacteria, the emergence of multi-resistant strains requires the study of new treatment methods. The aqueous extract obtained from the rhizome of *C. longa* demonstrated antimicrobial effects. The minimum inhibitory concentrations (MIC) obtained had values between 4 and 16 g/L, and the minimum bactericidal concentrations (MBC) varied between 16 and 32 g/L against *S. epidermis, Staph. aureus, Klebsiella pneumoniae* and *E. coli* [38,58]. Although the methanolic extract showed good inhibitory effects against *S. aureus* and *B. subtillis* the best results were obtained using hexanes and ethanol turmeric extract and curcuminoids against 24 bacterial species, namely *Vibrio harveyi, V. alginolyticus, V. vulnificus, V. parahaemolyticus, V. cholerae, Bacillus subtilis, B. cereus, Aeromonas hydrophila, Streptococcus agalactiae, Staphilococcus aureus, S. intermedius, S. epidermidis,* etc. [38,59]. A potential aim for antibacterials could be the stability and assembly of FtsZ protofilaments, an important factor for bacterial cytokinesis. By inducing, CCM abolished *B. subtilis* cytokinesis and Z-ring formation. A possible antibacterial mechanism of action produced by CCM is the inhibition of FtsZ assembly on the Z-ring and the suppression of bacterial cells [60].

In burn units, infections with *Pseudomonas aeruginosa* are the most common, and the multi-resistance to antibiotics acquired by this microorganism causes a major problem, complicating the treatment of patients. Shariati et al. (2019) used the wet-milling method to synthesize CCM nanoparticles with sizes between 2 and 40 nm. This large surface area enhanced aqueous solubility and produced better inhibitory effects on MDR *P. aeruginosa* than CCM extracts. These nanoparticles produced cell wall destruction, cell lysis, and death. It was demonstrated that nano-CCM produced a decrease in the number of genes responsible for the pathogenicity of the microorganism and the formation of biofilms. These effects were stronger than those caused by CCM due to the increased stability and solubility of nanoparticles [61].

Furthermore, the increase in the number of antibiotic-resistant strains led to research on the synergistic effect of antibiotics in combination with plant extracts. Combining CCM with antibiotics such as ampicillin, oxacillin, ciprofloxacin, and norfloxacin showed synergistic effects against the methicillin-resistant *S. aureus* strain, although the CCM-ciprofloxacin combination showed antagonistic effects against *S. typhi* and *S. typhimuriumin* [62,63].

### 2.3. Antifungal Effects

CCM also showed inhibitory effects on fungi. The methanolic extracts of *C. longa* showed inhibitory activity toward *Cryptococcus neoformans* (MIC-128 μg/mL) and *Candida albicans* (MIC-256 μg/mL), the hexane extract of the plant at 1 g/L was proved to have antifungal proprieties in the case of *Rhizoctonia solani, Phytophthora infestans,* and *Erysiphe graminis*, and the ethyl acetate extract, at the same concentration, had an inhibitory effect towards *R. solani, P. infestans, P. recondita,* and *Botrytis cinerea* [38,64].

An in vivo work on guinea pigs infected with *T. rubrum* confirmed that the cutaneous application of turmeric oil in a 1:80 dilution resulted in improved wound healing for a period of 2–5 days, and after 6–7 days the lesions disappeared [58]. Additionally, turmeric oil proved to be efficient against pathogens such as *Sporothrix schenckii*, *Exophiala jeanselmei*, *Fonsecaea pedrosoi* and *Scedosporium apiospermum* that produce inhibitory effects [38]. The foremost reason to examine the synergistic effect of CCM with the current biocidal chemical employed to eradicate parasitic fungi might be the potent antifungal properties of *C. longa* alongside its reduced side effects. The synergistic activity of CCM with several drugs was studied: voriconazole, itraconazole, ketoconazole, miconazole, nystatin, amphotericin B, and fluconazole (for the last two drugs, this synergistic activity could be understood by ROS accumulation [38]). These combinations exhibited a 10- to 35-time decrease in the MIC values of the fungicides toward 21 clinical isolates of *C. albicans* [65].

A plausible mechanism of cell death of *Candida* species induced by CCM treatment could be acidification of the intracellular environment by inhibiting H^+^ extrusion and targeting the global uptake suppressor thymidine 1, which resulted in the inhibition of hyphal development [65,66].

### 2.4. Antiviral Actions

Since most diseases caused by viruses do not have adequate treatments, or existing treatments are poorly tolerated or very expensive, researchers’ attention has turned to other treatment methods. Due to phytochemical compounds with multiple biological activities, plants were a subject of interest in these investigations [38]. Among the most important effects produced against viruses is the effect of CCM against HIV (human immunodeficiency virus). Due to effects on the function of some viral proteins, counting viral integrase, protease, and transactivator of transcription protein (Tat), CCM can inhibit HIV replication. CCM can inhibit viral integrase by directly interacting with the catalytic center of the protein and, if the Tat protein is considered, CCM prompts protein degradation at doses between 20 and 120 µM [40].

Curcumin-stabilized silver nanoparticles (CCM-AgNPs) have likewise been tested for their effectiveness toward HIV. When comparing the treatment of ACH-2 cells with CCM-AgNP with CCM alone, it was demonstrated that the nanoparticles were more effective in decreasing HIV-LTR expression even though CCM-AgNP did not entirely abolish the expression of HIV-1 p24 because the levels of the studied protein were constantly elevated during the infection. The expression of TNF-α (tumor necrosis factor alpha), interleukin-6, interleukin-1 β and nuclear factor kappa B decreased following CCM-AgNP treatment [67].

CCM has shown activity against the PR8, H1N1, and H6N1 influenza viruses. The results of the in vitro tests showed a more than 90% decrease in virus production in cell culture using low concentrations of CCM. Curcumin and its products such as gallium-curcumin and copper-curcumin have also produced antiviral effects towards other RNA viruses such as: Zika virus, by directly inhibiting cell attachment or inactivating the virus; Dengue virus, through indirect inhibition by influence on cellular systems rather than in a direct way on viral functions; and HCV, by decreasing replication by overturning the Akt-SREBP-1 path [58]. Against the hepatitis B virus, the *C. longa* extract blocked its replication by rising the rate of p53 protein by improving protein stability and activating the transcription of the p53 gene [38].

Human papillomaviruses (HPV) are also responsible for the appearance of cervical cancer, the viral oncoproteins E6 and E7 being responsible for this. CCM had a hindering activity towards the expressed genes of the E6 and E7 proteins of HPV-16 and HPV-18 as two of the main oncogenic HPV [68]. CCM alongside its analogs is able to inhibit the replication of numerous viruses through various mechanisms. However, because it is rapidly metabolized, CCM has low bioavailability, thus reducing curcumin efficiency as an antiviral compound [40].

### 2.5. Antioxidant and Anti-Inflammatory Action of Curcumin

**Figure 4:**
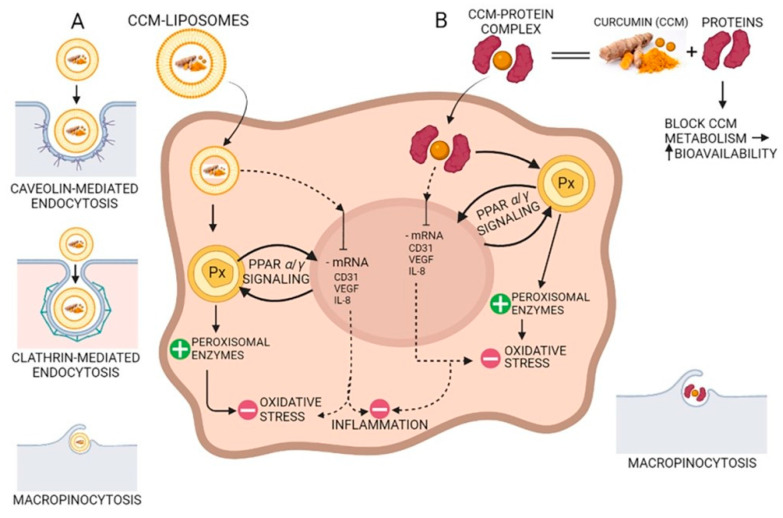
The antioxidant and anti-inflammatory action of curcumin (CCM). (**A**) CCM-liposomes are internalized in the cell by three routes: (i) caveolin-mediated or (ii) clathrin-mediated endocytosis, and also (iii) macropinocytosis. CCM targets the PPARα/γ (peroxisome proliferator-activated receptor alpha/gamma) signaling and potentiates peroxisome activity by increasing peroxisomal enzymes (e.g., catalase, superoxide-dismutase). Increasing the activity of peroxisomal-derived antioxidant enzymes leads to a reduction in reactive oxidative species (ROS) and a decrease in oxidative stress. Additionally, CCM inhibits mRNA of CD31 (cluster of differentiation 31), IL-8 (interleukin 8), and VEGF (vascular endothelial growth factor) with anti-inflammatory effects in the cell. (**B**) Complexes of CCM-proteins act on the peroxisome by increasing peroxisomal enzymes, thus decreasing oxidative stress by their scavenging action on the ROS. Furthermore, like CCM internalized by caveolin/clathrin-mediated endocytosis, the CCM-protein complex inhibits mRNA of CD31, IL-8, and VEGF, thereby demonstrating anti-inflammatory activity due to CCM increased bioavailability.

Due to the hydroxyl and methoxy groups, CCM can have antioxidant and anti-inflammatory effects. Acting on different molecular targets such as interleukins and cytokines, CCM inhibits the proliferation of inflammatory cells, metastases, and angiogenesis via CD31 (cluster of differentiation 31), VEGF (vein endothelial growth factor), and IL-8 (interleukin 8) signaling as shown in Figure 4. CCM has proven to be efficient in the management of several inflammatory illnesses such as diabetes, obesity, and various neurological and cardiovascular diseases [69]. Many inflammatory transcription factors, cytokines, and enzymes play a significant part in the onset and evolution of type II diabetes and the main cause of the development is inflammation. CCM can increase antihyperglycemic and insulin sensitivity by reducing glucose produced in the liver, decreasing the inflammatory response arising from hyperglycemia, increasing the expression of GLUT2, GLUT3, and GLUT4 genes, cellular glucose uptake, and activating AMPK [70]. CCM increases adiponectin production, and decreases macrophage infiltration, leptin, and leptin receptor levels in the case of inflammation-related obesity. The increase in adiponectin production leads to a decrease in NF-kB activity, having a positive effect against obesity [69]. CCM treatment activates the Nrf2-dependent antioxidant response, inhibits TNF-α in blood vessel smooth muscle cells, and increases p21 expression. A study conducted on individuals suffering from coronary heart disease proved that CCM can lower serum levels of triglycerides, LDL, and VLDL cholesterol, although no significant effects on inflammatory markers were observed [71]. New aspects about the antioxidant effect of curcumin suggest that CCM stimulates the peroxisomal enzymes such as catalase, as a prominent antioxidant factor reducing the oxidative stress via the PPRA α/γ (peroxisome proliferator-activated receptor alfa/gamma) pathway (Figure 4).

Curcumin also has a role in the treatment of some neurodegenerative disorders caused by oxidative or inflammatory effects. In the case of Alzheimer’s disease, CCM acts as an antioxidant, improving cognitive functions and delaying neuronal damage. Due to CCM’s inhibitory effect on inflammatory cytokines and the associated JAK-STAT, AP-1 and NF-kB signaling pathways, it can be used in the treatment of multiple sclerosis, a chronic inflammatory autoimmune disease that results in the degradation of the myelin sheath of neurons. CCM also suppresses Th17 cell differentiation, which is a significant factor in the development of multiple sclerosis [69].

### 2.6. Antitumoral Effects of Curcumin

Curcumin has antitumor effects affecting two primary processes: tumor growth and angiogenesis. CCM has effects against angiogenesis through different mechanisms: acting on transcription factors NF-kB and AP-1; limiting the expression of IL-8 in pancreatic cancer and head and neck cancer cell lines; inhibiting COX-2 (cyclo-oxygenase-2) and 5-LOX (5-lipo-oxygenase); inhibiting angiogenesis mediated by NO and iNOS (inducible nitric oxide synthase). CCM induces cell death in several cancer cell lines, including melanoma, leukemia, lung, colon, ovarian, liver, and breast carcinomas. The induction of apoptosis is linked to the action of CCM on the proteasome. In small doses, CCM activates the proteasome leading to cell survival, while in high doses, it inhibits the proteasome, leading to apoptosis. In addition, the dose of CCM can affect the type of cell death. High doses reduce the fabrication of reactive oxygen species and cause cell death through necrosis, while small doses result in oxidative stress and apoptosis [72].

An in vivo study performed in mice receiving a brain bolus of B16F10 melanoma cells showed that CCM effectively inhibited P-NF-kB, BclXL, cyclin D1, P-Akt and VEGF, elucidating its effectiveness in blocking proliferation, survival, and invasion of B16F10 cells in the nervous system [22].

A study carried out in Wistar rats showed that CCM prevents the formation of hyperplastic liver nodules [73]. Microcapsules containing curcumin exhibited high, dose-dependent cytotoxicity on HCT-116 colorectal carcinoma cell culture [74]. Composites containing curcumin showed increased cytotoxicity in human breast carcinoma (MCF-7) and human lung adenocarcinoma (A549) [75]. Topical application of CCM significantly inhibited the formation of papilloma in female CD-1 mice [76].

**Figure 5:**
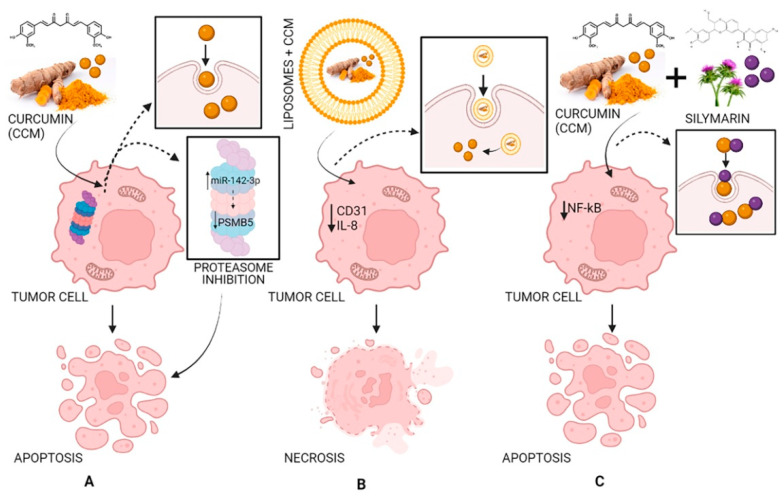
The antitumor activity of CCM (**A**), of liposomes loaded with CCM (**B**), and of CCM complexes with silymarin (**C**). Strategy and pathway (**A**): CCM inhibits the proteasome by increasing miR-142–3p expression leading to negatively regulating PSMB5 (proteasome 20S subunit beta 5) leading to cell apoptosis; strategy and pathway (**B**): Liposomes loaded with CCM decrease the expression of CD31 (cluster of differentiation 31) and IL-8 (interleukin 8) leading to cell necrosis; strategy and pathway (**C**): CCM complexes with silymarin inhibit NF-κB (nuclear factor-κB) leading to cell apoptosis.

In a xenograft model study, pancreatic cancer cells were injected subcutaneously into female nude mice. It was demonstrated that liposomes loaded with CCM can have antitumor effects by decreasing the expression of CD31 as well as that of VEGF and IL-8, demonstrating that CCM blocked the evolution of pancreatic carcinoma in these types of models and inhibited tumor angiogenesis (Figure 5) [72,77,78].

## 3. Curcumin Bioavailability Improving

In the last decade, researchers have focused on trying to optimize the beneficial properties of CCM and improve its pharmacokinetic profile. To increase the stability and solubility of CCM, plenty of methods are described in the literature, such as the addition of human serum albumin [79] or other types of proteins [80,81], incorporation into oil-in-water emulsions [82], cyclodextrin inclusion complexes [83], or by using solid dispersion technology [84,85].

### 3.1. Curcumin-Based Solid Dispersions

**Figure 6:**
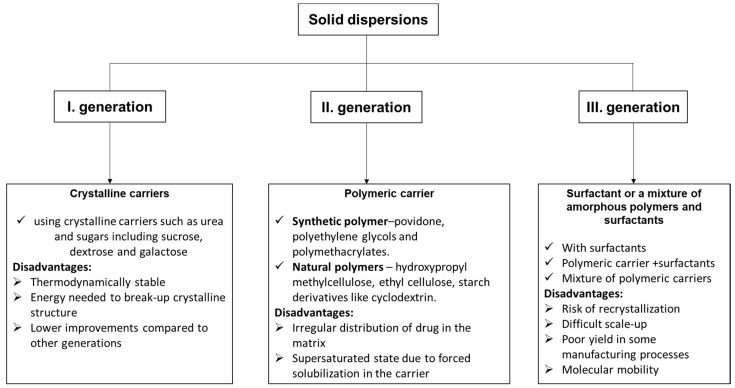
The types of solid dispersions [89] and their disadvantages.

Solid dispersion is one of the most extensively and efficaciously applied methods in the medical field for improving the solubility and dissolution rates, and thus the bioavailability of molecules with low solubility. It is founded on the idea that the hydrophobic molecule is dispersed in an inert carrier that has the property of being soluble in water in the solid state [86]. Depending on the carriers used, solid dispersions (SD) have three categories [87]: first-generation crystalline solid dispersions, as their name suggests, which are crystalline carriers. Among the most used, we mention urea, sucrose, dextrose, and galactose, resulting in a long release of drugs (Figure 6). They present the disadvantage that they form crystalline solid dispersions (more stable forms from a thermodynamic point of view), and also release the medicine more slowly than the amorphous ones. In the second generation (SDs), the active molecule is typically dispersed in a non-crystalline solid carrier (amorphous polymer). This second class can be classified taking into account the molecular interaction that takes place between the drug and carriers in solid solutions or suspensions or a combination of both. Depending on the origin of the polymer used, the dispersions are classified into fully synthetic polymers (povidone, polyethylene glycols, and polymethacrylates) and natural product-based polymers (hydroxypropyl methylcellulose, ethyl cellulose, and starch products such as cyclodextrin).

The irregular distribution of drugs in the matrix and the supersaturated state due to forced solubilization in the carrier are disadvantages of these dispersions. In the third generation, the active molecules are dispersed in a surfactant or in a blend of amorphous polymers and surfactants. The bioavailability of a low water-soluble drug was significantly better by using a combination of water-soluble polymer and surfactants. The resulting dispersions were physically and chemically stable for no less than 16 months [88]. The main drawback of these III generation solid dispersions is their reduced suitability for industrial production.

In the literature there are numerous studies that use polyethylene glycols as carriers for second- and third-generation solid dispersions. During the PEGylation process, covalent or non-covalent attachment or combination of the CCM molecules to the PEG chains takes place [90,91]. PEG is one of the most many-sided, biocompatible polymers permitted by the FDA as a carrier or base for several pharmaceutical preparations with reduced toxicity [92,93]. There are three methods to create a solid dispersion: *the melting method* (the hydrophobic molecule is dissolved in a liquid and the resulting solution is added to the melted PEG, without evaporation of the solvent)*, the melt agglomeration method* (by adding the molten carrier comprising the hydrophobic molecule to the heated excipients), and *the solvent evaporation method* (by dissolution of the hydrophobic molecule and carrier in a volatile solvent followed by its evaporation) [94]. The last two described methods are the main procedures for obtaining solid dispersions.

Based on the study by Mittal et al. (2019) [95], PEGylated CCM nanoliposomes with 1000-fold enhanced CCM hydrophilicity and ten-fold higher stability were prepared, as well as in vitro release kinetics indicating two-fold higher CCM release in the simulated gastric and intestinal environment. A study by Li et al. (2009) [96] revealed that PEGylated CCM inhibited cell proliferation in pancreatic cancer cells and showed a superior result in decreased proliferation when compared to CCM alone. A good example of third-generation solid dispersion is poly-(lactic acid) (PLA) and PEG amphiphilic copolymers for CCM encapsulation. PEG is a hydrophilic surface that can mask the hydrophobic core of PLA, which is a portal for CCM. In the study of Liang et al. (2016), it is pointed out that three-generation dispersion (PLA-PEG) can be used for the delivery of CCM in the desired therapeutic amount to the target cells [97]. Another example is presented in the research of Khalil et al. (2013) [98], wherein they discovered that in a polylactic-co-glycolic acid and PLGA-polyethylene glycol mixture, nanoparticles containing CCM increased the CCM bioavailability by approximately 15 and 55-times when compared to a water suspension of CCM in rats. Yildiz et al. (2019), by employing polyethylene glycol mono-oleate-460 for the oily phase, increased CCM solubility by approximately 710 times compared to raw CCM, and CCM also remained chemically stable in emulsion [99].

### 3.2. Curcumin Complexes with Proteins

Complexation with proteins has been described as an encouraging technique to increase CCM bioavailability and solubility in aqueous solutions, and can improve the unfavorable pharmacokinetic and antioxidant properties of CCM [100,101]. Protein-based carriers have been widely used in the field of biomedicine nanoparticles [102]. When curcumin, hydrophobic in nature, is mixed with an amphiphilic protein in an aqueous environment, the said protein suffers a self-assembly process. Here, the interior of the protein traps the hydrophobic core, and the hydrophilic surface forms a micelle structure [81].

There are numerous studies that reveal the effects of added protein on the properties of CCM through complexation with milk proteins, for example, casein [103], whey protein isolate (WPI) [104], bovine serum albumin [105], or with plant-derived proteins such as sunflower protein isolate [106], mung bean protein [107], and soy protein [108,109].

In all the studies presented, plant-origin proteins have a high capacity to load water-insoluble CCM into hydrophobic sites and improved the water solubility and stability of CCM by complexation [107,108,109]. Proteins extracted from milk (whey and casein) [110] have the property of being able to be used as carriers of hydrophobic molecules/ions. They are also very good interfacial agents, which means that they are employed in the preparation and stabilization of emulsions that contain hydrophobic molecules [111]. Caseins, which are found in natural milk as supramolecular aggregates (casein micelles), are the main milk proteins and have outstanding emulsification, gelation, and water-binding properties [112,113]. CCM generally binds to caseins in milk; nevertheless, whey protein plays a part in the binding affinity [103,112]. Whey protein (WP) is found in three forms: (i) concentrate (WPC), (ii) isolate (WPI), and (iii) hydrolyzed (HWP). WPC consists of 80% protein and 20% fat and sugar (carbohydrates, lactose), while in WPI, the protein content is more than 90% [114] and comes from the ultrafiltration and diafiltration processes during cheese manufacturing [115,116]. The main constituents of WPI are globular proteins comprising beta-lactoglobulin, alpha-lactalbumin, and bovine serum albumin [117]. These proteins have the property to make transparent and flexible comestible films that are an obstacle to aroma at a small relative humidity. However, WPI films present low mechanical characteristics and offer a low moisture obstacle due to their hydrophilic features [118,119]. As a solution to this problem, the incorporation of phenolic substances was tried as a method to better the moisture barrier and bio-functional characteristics of these films [120]. Due to its nutritional properties and various technological functionalities, whey proteins are broadly used in different food products and as several kinds of nutraceutical-carrying systems [121]. Taking into account several factors, for example, the concentration of the primary protein, the temperature, and the pH at which the process takes place, but also the ionic strength, whey proteins can take different structures, for example, aggregates in various forms, nano-fibrils/tubes/particles, flexible fibers, and microgels/particles [122]. From this class of proteins, particularly ß-lactoglobulin has the ability to self-assemble, forming nanofibrillar aggregates of a length comprised between 1 and 10 μm with a diameter found in the nano range (1–10 nm) by long thermal treatment under very acidic conditions (pH approximately 2) and small ionic strength [123]. Whey protein nanofibrils have enhanced techno-functional characteristics when paralleled to the non-fibrillate ones. They have the ability to create self-supporting hydrogels at small protein concentrations, have superior emulsifying and foaming characteristics, and have a greater ability to increase viscosity [124,125,126]. All these were described in an original work by Mohammadian et al. 2019 [100], who used whey protein nanofibrils as carriers in order to increase the water dispersibility of CCM.

When the CCM forms a complex with nanofibrils (pH 3.2) the solubility in water increases considerably while the sedimentation over time is declining. Additionally, these complexes present a great affinity towards interfaces and great viscosity that could be utilized in the food industry to produce new functional foods. Another way to transport CCM is to make an emulsion loaded with CCM obtained from proteins or polysaccharides, such as soy carbohydrates or casein, whey protein, Arabic gum, lecithin, Tween-80, etc. [127,128]. Regrettably, these complexes have not proved very stable, which is why they disintegrate over time, affecting their storage [129,130,131,132]. In a recent study by Ye et al. (2021) [133], it was demonstrated that the swing of WPI conformation owing to desolvation can be kept by spray drying. A recent review [134] examined whether the complexation among proteins and small molecules that have hydrophobic properties in desolvation might likewise be kept in the microparticles following spray drying. Therefore, the microparticles were combined in yogurt to observe the effects on targeted release, bioavailability, and sensory properties. The results of the study highlighted the fact that even if the existence of yogurt marks the discharge of CCM from the complex, the kinetic characteristics (burst or sustained) release persist, thus emphasizing the part of food constituents in bioavailability [135].

Thus, it was demonstrated that proteins such as zein, ovotransferrin, whey protein isolate, whey protein nanogel, and casein display probable properties for stabilization of CCM emulsions, and if the complexation is done with acids, or other polysaccharides, their surface wettability can be improved. It is important to enhance CCM bioavailability during intestinal absorption by enhancing its stability against degradation [136]. In this sense, it is known that whey proteins have the capability to form cold-set hydrogels. This technique named cold-set gelation involves two consecutive stages: (i) obtaining the soluble aggregates by heating a protein solution and (ii) cooling of the obtained solution followed by salt-induced cold gelation (adding CaCl_2_ and NaCl) or acid-induced cold gelatins (adding glucono-D-lactone) [137]. Cold-set gels, compared with to heat-set gels, have the ability to transport temperature-sensitive bioceuticals, such as thiamine (vitamin B1) and probiotics. A recent gelation method developed by Alavi et al. (2018) [137] is centered on the radical cross-linking of proteins. This method removes the thermal treatment stage in the process of obtaining water-soluble aggregates thus obtaining the gelation of non-heat-treated whey proteins. Due to poor mechanical resistance, the cold-set whey protein hydrogels might be susceptible to enzymatic degradation. For prevention, a solution would be to obtain gels based on proteins and polysaccharides that give them the properties to remain intact in some areas of the superior gastrointestinal tract, and afterward disintegrate and deliver the active ingredient in the colon [138,139].

Due to these properties, glycation of the whey protein isolate with glucose might lead to an increase in the bioavailability of CCM [136]. Thus, in a recent study, an attempt was made to create a mixed hydrogel consisting of whey protein aggregates (WPA)/k-carrageen using a designed microstructure to shield CCM inside the superior gastrointestinal tract and transport it to the colon. In this way, this combination was demonstrated to have a great ability to load CCM, but at the same time prevent the loaded CCM from discharge and being destroyed in this way in the gastrointestinal tract. In this case, it was validated that hydrogels are appropriate for the colon-specific delivery of nutraceuticals [140].

As mentioned above, WP include β-lactoglobulin (β-Lg), α-lactalbumin (α-La), immunoglobulins (IG), bovine serum albumin (BSA), bovine lactoferrin (BLF), and lactoperoxidase (LP), together with other minor components [141]. The central compounds of whey protein concentrate are β-lactoglobulin (β-Lg) and α-lactoglobulin, which can interact with CCM. More than 50% of the whey proteins in mammal milk consist of β-Lg, a member of the lipocalin family of proteins, which comprises a single polypeptide of 162 amino acids with a total molecular weight of 18.4 kDa and has the capability to bind small hydrophobic molecules into a hydrophobic cavity [142]. It is accepted that β-Lg is one of the best natural polymer matrices owing to its amazing nutritional value, powerful binding affinity, and resistance to pepsin hydrolysis, which makes it one of the main delivery vectors [143]. Β-Lg also has 2 tryptophan (Trp) residues, Trp 19, located in a hydrophobic pocket, and Trp 61, located on the surface of the protein close to the pocket [144]. β-lactoglobulin was a compact globular structure, due to this fact, it has great resistance to breakdown in the gastric environment [145]. Curcumin molecules can not only be easily connected on the surface of proteins through a facile complexation method [112], but also be incorporated into the interior side of protein, because the structure of a protein is highly dependent on the pH medium used [146]. Fluorescence measurements, Raman spectroscopy, and Fourier transform infra-red (FTIR) spectroscopy have shown that the complexation took place through the hydrogen bonding and hydrophobic interactions between CCM and proteins [147]. Circular dichroism (CD) spectroscopy and X-ray diffraction (XRD) analysis revealed the secondary structures and crystallinity of the protein [148] and scanning electron microscopy (SEM) investigated the morphological properties of the prepared complexes. CD represents a very good instrument for the fast determination of the secondary structure and folding properties of proteins [149].

#### 3.2.1. Using Fluorescence Spectroscopy to Monitor the Interaction between CCM and Proteins

Fluorescence spectroscopy represents a powerful method for studying the interactions between proteins and active substances. Proteins have inherent fluorescence due to the existence of aromatic amino acid residues in their structure. The fluorescence quenching of proteins by CCM can be considered to be in a static quenching. The biomolecular quenching constant (Kq) is greater than the limiting diffusion rate constant of the biomolecule (2.0 × 10^10^ L mol^−1^s^−1^) [150].

When curcumin is bound to proteins in the static mode of fluorescence quenching, the binding reaction is nearly irreversible, while in the case of dynamic quenching, there is a dynamic balance between association and dissociation of CCM-protein complexes [151]. The binding parameters such as the binding number (n) and equilibrium association constant (K_a_) can be calculated according to the following equation (Equation (1)) [101,152]:

(1) $$\log\frac{F_{0}-F}{F}={\mathrm{logK}}_{a}+n\log\left[\mathrm{CCM}\right]$$

where K_a_ is the equilibrium association constant (L/mol), F_0_ represents the fluorescence intensity without quencher, e.g., curcumin is CCM, F is the fluorescence intensity in the presence of quencher, and [CCM] is the concentration of quencher.

In addition to the binding parameters, the change in enthalpy ($\Delta H^{o}$) and entropy ($\Delta S^{o}$) can be calculated from Van’t Hoff equation (Equation (2)), by plotting lnK_a_ versus 1/T (T is the absolute temperature):

(2) $${\ln\;K}_{a}=-\frac{\Delta H^{o}}{\mathrm{RT}}+\frac{\Delta S^{o}}{R}$$

where K_a_ is the equilibrium association constant, at the corresponding temperature (T in K) and R is the gas constant [J/(mol·K)]. The $\Delta H^{o}$ and $\Delta S^{o}$ were determined, respectively, from the slope and the y-intercept of Van’t Hoff linearization: $\ln\;K_{a}$ = f(1/T). The free energy change ($\Delta G^{o}$) was calculated by Gibbs–Helmholtz (Equation (3)):

(3) $$\Delta G^{o}=\Delta H^{o}-T\Delta S^{o}$$

Although curcumin might be of help for human health benefits due to its high antioxidant and anti-inflammatory properties, there is a limitation in its use due to its low solubility and stability, and, consequently, a better understanding of its absorption and metabolism is needed to properly take advantage of its potential health impact. Low absorption of CCM in the small intestine leads to its low oral bioavailability. It is known that there is a tendency for CCM to bind to structure-modifying enterocyte proteins (intestinal adsorptive cells) [153]. Thus, it is plausible to hypothesize that a promising solution to enhance low CCM bioavailability comes in the form of protein-based complexation or encapsulation of CCM with different types of proteins, both animal- and plant-based ones that are already being studied.

Protein from whey is arguably the most researched type of protein in relation to curcumin, in the form of whey protein isolate (WPI) [154,155,156,157,158], as well as whey protein concentrate (WPC) [158,159,160,161,162,163,164]. However, while both WPI and WPC come from whey, there is a significant difference between them in terms of processing and final product composition. As such, the highly-processed isolate form has a much higher protein content (at least 90%), containing fewer fats, lactose, and carbohydrates. In turn, the concentrated form, while containing less protein (34–85%), has a much lower cost, making its extended use in commercial dietary supplements understandable.

However, the fact that whey protein has many different components needs to be taken into account as they might bind differently to curcumin. Furthermore, the fact that the protein portion of whey consists of many distinct proteins, such as β-lactoglobulin, α-lactalbumin, bovine serum albumin (BSA), immunoglobulins, lactoferrin, lactoperoxidase, and glycomacropeptide, must also be considered. There is considerable literature that focuses on the interactions of these individual proteins and curcumin [165,166,167,168,169,170,171,172,173,174], for example, CCM and β-lactoglobulin [158,165,166,167,168,169], α-lactalbumin [170,173], bovine serum albumin [171,172], or lactoferrin [174].

Casein, the other milk protein, while slower than whey in terms of amino-acid absorption by the body, has also been studied with respect to curcumin complexation [158,161,175].

Alongside all these milk-derived proteins, plant-based ones such as soy [176,177,178,179,180,181], pea [182], walnut [183], rice [184], or zein [185,186,187,188,189,190] have also been researched as carriers or in complexes with CCM. Although hydrophobic interactions are widely accepted to play a primary role in the CCM-protein complexation process [191,192,193,194,195,196,197,198], more studies are still required for a better understanding of nano-complexation and the design of new complexes with enhanced stability of curcumin in vitro and in vivo.

In Table 1, the results presented in various recent articles from the state of the art are summarized. The quenching mechanism is static in almost all cases. Table 1 also provides some thermodynamic parameters from a selection of more recent and representative studies that employ a wide array of animal- and plant-derived proteins. For example, different types of proteins (see Table 1) have been studied in relation to CCM complexation, primarily in binary systems and occasionally in systems with multiple other components [101,171,172,180,182,184,191,192,193,196,198].

Table 1 presents a summary of the thermodynamic approach for complexes of CCM and different proteins, derived from fluorescence measurements. Considering the importance of thermodynamic parameters and protein–curcumin affinity in increasing CCM bioavailability by using proteins, the binding constant (either K_S_, K_a_ or K_b_ as noted in original works) and the Stern-Volmer constant (K_SV_) were chosen for comparison of data listed in Table 1. From these data, it should be noted that curcumin binds in a similar manner to different types of proteins.

For instance, curcumin interacts with β-lactoglobulin, at pH 7, with a binding constant, K_S_ of 1.33 × 10^5^ LM^−1^, to form the 1:1 complex of CCM-β-Lg at room temperature [101], (about 20 °C), and the corresponding free energy change (e.g., $\Delta G^{o}$ = −RT lnK_S_) is about −28.7 kJ/mol. Similar results are reported for the binding (association) constant of curcumin, K_a_ of 0.119 × 10^5^ LM^−1^ (at 25 °C, pH 7.4) to β-Lg [196], in the same order of 10^5^, and $\Delta G^{o}$ is about −23.2 kJ/mol. The difference between these values is supported by the different experimental conditions, given that the protein conformation depends on the pH and temperature.

Furthermore, different proteins might bind differently to curcumin, as observed in Table 1, where a wide variety of proteins, either animal or plant-based, are studied in interaction with curcumin.

This observation is in good agreement with the binding of curcumin to bovine serum albumin, either K_a_: (0.724 ± 0.01) × 10^5^ L/mol and n = 1.110 ± 0.038 (at 298 K and pH 7.4 as given in [171]), or K_b_: 1.95 × 10^5^ L·mol^−1^ and n = 1.11 (at 25 °C and pH 7.0 in [172]), as well curcumin with human serum albumin (at 298 K, pH 7.4 in [191), both K_b_: (17.3 ± 0.32) × 10^6^ M^−1^ and n = 1), for the CCM-HSA complex, and curcumin with holo-transferrin, K_b_: (1.81 ± 0.32) × 10^6^ M^−1^) for the CCM-HTR complex, or K_b_: (3.19 ± 0.32) × 10^6^ M^−1^ for the ternary CCM-HSA-HTR complex. Apparently, the interaction of CCM with human serum albumin [191] is rather higher than its interaction with bovine serum albumin [171,172]. Moreover, the free energy changes, $\Delta G^{o}$, for the interaction of CCM with serum albumins are in the same order of tens, namely −23.05 ± 0.02 kJ/mol, for the CCM-BSA complex [171], and −30.19 kJ/mol for the CCM-BSA complex [172], as well as −41.29 kJ·mol^−1^, for the CCM-HSA complex, −36.46 kJ·mol^−1^ for the CCM-HTR complex, and −37.98 kJ·mol^−1^, for the ternary CCM-HSA-HTR complex, as described in [191].

The interaction of curcumin with whey protein isolate is characterized by, K_a_: 10.8 × 10^5^ L·mol^−1^ and n = 1.30 (for 308 K and pH 7.0) as shown in [192], curcumin with β-casein binding by K_b_: (0.398 ± 0.26) × 10^5^ L·mol^−1^ and n = 1.59 ± 0.14 (at 298 K and pH 7.4) as given in [193], and curcumin with bovine lactoferrin by K_b_: (0.301 ± 0.16) × 10^5^ M^−1^ and n = 1.03 ± 0.16 (at 299.15 K and pH 7.4 [198]). The free energy changes, $\Delta G^{o}$, for the interaction of CCM with WPI is −37.08 kJ·mol^−1^, for the CCM-WPI complex [192], −26.2 ± 0.09 kJ·mol^−1^ for the curcumin and β-casein complex [193], and −25.65 kJ·mol^−1^ for the curcumin and bovine lactoferrin complex [198].

The binding of curcumin to the pea protein isolate is characterized by K_a_: 0.474 × 10^5^ L/M, and n = 0.97 (at 298 K, pH 7.0) in [182] and K_a_: 76.8 × 10^5^ L·mol^−1^ and n = 1.44 (at 308 K, pH 7.0) in [192]. The free energy changes, $\Delta G^{o}$, for the interaction of CCM with PPI is −26.67 kJ·M^−1^, for the CCM-PPI complex [182], and −41.00 kJ·mol^−1^ for the CCM-PPI complex [192]. These results are justified by the significant difference in temperature, K_a_ and $\Delta G^{o}$ are higher at 308 [192] than at 298 K [182]. The binding of curcumin to soy protein is shown as K_SV_: 0.63 × 10^5^ M^−1^ (at room temperature and pH 12.0) and $\Delta G^{o}$: −26.90 kJ⋅mol^−1^ in [180] and similarly for the curcumin and rice protein complex, K_SV_: 2.17 × 10^5^ M^−1^ and $\Delta G^{o}$: −30.3 kJ/mol (for 298 K and pH 12.0) are also given in [184].

Similar results were observed versus other pHs and temperatures [101,171,172,180,182,184,191,192,193,196,198] and the CCM-protein complexes are mainly studied for the 1:1 molar ratio, showing that one binding position is available in each type of protein. However, the mixture of protein-based nano-carriers could be of great potential to deliver more than one bioactive compound simultaneously for drug delivery systems or for functionalized hydrocolloids in the food industry.

Therefore, it is important to characterize and understand every system at the molecular level. For this purpose, several characterization methods can be employed, such as circular dichroism (CD) spectroscopy, Fourier transform infra-red (FTIR) spectroscopy, isothermal titration calorimetry (ITC), electrospray ionization mass spectrometry (EIMS), nuclear magnetic resonance (NMR), or maybe the most widely used one, fluorescence quenching. The results of the fluorescence measurements can be used to estimate the binding parameters of curcumin to the protein and the binding mechanism.

Depending on the medium used, the binding constant varies between 1.19 × 10^4^ and 1.1 × 10^5^ M^−1^ between CCM and β-lactoglobulin (β-Lg) and hydrophobic interactions play a major role in their binding. In the case of using bovine serum albumin, the quenching mechanism is also static, and the values of binding constant are between 1.6 × 10^4^–2 × 10^5^ M^−1^ depending on the used medium, and the binding of CCM to BSA was driven by entropy. In all cases, the binding constants suggest a great ability to form complexes with CCM [150].

#### 3.2.2. Using Circular Dichroism for Monitoring the Protein’s Secondary Structure

Circular dichroism (CD) is a chiroptical method to study the binding of CCM to biomacromolecules, including proteins or polysaccharides [194]. In order to determine precisely the secondary structure of a protein using CD data, the obtained results need to comprise a spectral range between 240 and 190 nm. Furthermore, these analyses are considerably affected by the precision of the protein concentration measurement, since this will influence the magnitude of the CD spectrum when scaled to standard units.

In the study by Mohammadian et al. (2019) [100], the authors used whey protein nanofibrils (WPN) to improve curcumin’s solubility. They showed that the solubility was improved by 1200-fold, while complexation with WPI improved its solubility by around 180-fold when compared with the native CCM. CD spectroscopy showed that the secondary structures of WPN were not meaningfully touched by binding to CCM.

The CD spectrum of WPI exhibited two troughs at wavelengths of around 218 and 210 nm, which are typical for β-sheet and α-helical structures related to the main fractions of whey proteins including β-Lg and α-La. The binding of β-Lg to CCM at a pH value of 7.0 amplified the contents of the α-3 helix, β-sheet, and β-turn.

#### 3.2.3. Molecular Docking for Modelling the Binding between Curcumin and Protein

Currently, molecular docking has a significant role in structure-based drug design [195]. The method allows one to describe the comportment of small molecules in the binding site of target proteins [152,182], and also to clarify fundamental biochemical processes [196]. The procedure includes two phases: (i) the prediction of the ligand conformation and its position and orientation within these sites and (ii) the assessment of the binding affinity [193]. In molecular docking, thousands of possible binding sites and modes of binding among proteins and ligands are identified automatically by the software.

This technique is auxiliary, but is essential to describe the interaction among proteins and CCM, which would help to explain the results obtained using other techniques (Table 1).

#### 3.2.4. Infrared Spectroscopy

Infrared (IR) spectroscopy is a firmly-established investigational technique for the examination of the secondary structure of proteins. Amide I (peak position arises in the region of 1700–1600 cm^−1^-mostly C=O stretch) and II bands (peak position arises in the region of 1600–1500 cm^−1^-C-N stretch coupled with N-H bending mode) represent very significant IR absorption bands to characterize the secondary structure of the protein [197]. The secondary structural compositions of the free protein and of the protein in its CCM complexes were calculated by FTIR difference spectra, self-deconvolution, and curve-fitting procedures of the amide I band. The resultant fitted curve was analyzed and each band was given to a secondary structure taking into account the frequency of its maximum; β-antiparallel: 1684–1696 cm^−1^; turn: 1662–1679 cm^−1^; β-sheet: 1620–1638 cm^−1^; the remainder mainly includes α-helix: 1654–1659 cm^−1^ and random coil: 1640–1648 cm^−1^. The variation of these bands predicts the complex CCM-protein formation and the existence of binding interactions between the small molecules (such as CCM) and proteins [198].

### 3.3. Combination with Bioactive Molecules

**Figure 7:**
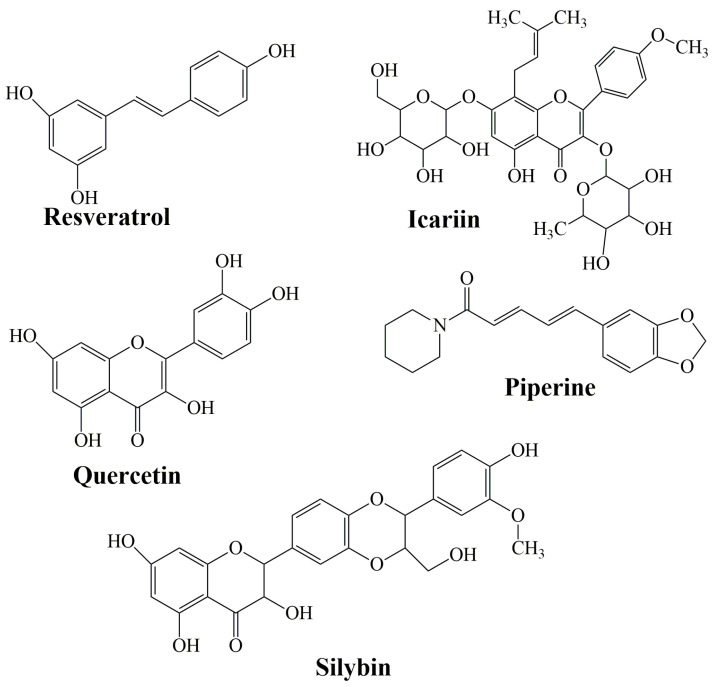
The structure of resveratrol, icariin, quercetin, piperine, and silybin as representative bioactive molecules.

Flavonoids are a family of compounds including flavonols, flavanones, isoflavones, and flavans. Flavonoids are known as the characteristic color anthocyanin pigments of plant tissues, which possess various bioactivities such as anti-inflammatory, analgesic, anti-diabetic and anticancer effects [199,200]. Some studies have shown that a treatment that combines curcumin equivalents with other therapeutic agents has better efficacy compared to monotherapy [201,202]. Montgomery et al. (2016) [203] revealed that the combined use of CCM and silymarin is 5-fold more effective against colorectal cancer compared to cells treated with silymarin. *Silymarin* (SIL) is a group of flavonoids that has been isolated from the fruits of *Silybum marianum* [204]. The main active ingredient in silymarin is silybin (silibin) and silibinin (Figure 7) [205].

Historically, SIL has been used medicinally to treat disorders of the liver, spleen, and gallbladder. It has also been found to be effective in the treatment of breast, prostate, and skin cancers [206]. The combination of SIL with CCM revealed that it is more effective and efficient in radiation-induced kidney injury than CCM or SIL alone, so it could be used to protect patients during radiotherapy [207]. This effect could be further enhanced by the complexation of curcumin with protein [208] or by complexation of beta-cyclodextrins with other different bioactive molecules such as curcumin [209], quercetin [210], lipoic acid [211,212], or deferoxamine [213].

*Icariin* (ICA) (Figure 7) is a flavonol glycoside extracted from the *Epimedium* genus which has been employed in traditional Chinese medicine since ancient times [214]. It has been described to have an extensive variety of biological and pharmacological effects [215]. It decrees the levels of total serum cholesterol and low-density lipoprotein cholesterol [216], promotes the differentiation and proliferation of osteoblast, and inhibits human prostate carcinoma and the proliferation of breast cancer cells [217].

*Quercetin* (QRC) [2-(3,4-dihydroxyphenyl)-3,5,7-trihydroxy-4H-chromen-4-one] (Figure 7) is a plant-derived flavonoid, its richest source is onions, curly kale, leeks, broccoli, and blueberries [218]. QRC has antioxidant, anti-inflammatory, and anticancer properties [219]. The synergistic use of QRC and CCM as an antitumor drug is stronger than individual treatment [220]. In the study of Altundag et al. (2021) [221], the authors revealed that the combination treatment of these was effective against chronic myeloid leukemia cancer cells without affecting healthy cells. Co-delivery of natural substances such as piperine, quercetin, resveratrol, and silibinin reduced the metabolism of CCM which leads to a rise in CCM absorption [222].

*Resveratrol* (Res) (3,5,4′-trihydroxystilbene) is a naturally arising polyphenol that has been reported as a cardioprotective, neuroprotective, and chemo-preventive agent, along with antiaging properties [223,224,225]. Dietary administration of CCM with resveratrol against prostate, neck, liver, and head cancer had shown a significant decrease in tumor growth [226,227,228]. Res obtained the highest improvement in permeability when mixed with other agents: quercetin (310%), CCM (300%), quercetin, and CCM (323%, 350% with piperine).

*Piperine* (PIP) (Figure 7) is an alkaloid substance found in black pepper (*P. nigrum*). It was proven that PIP behaves as a bio-enhancer of several drugs and inhibits drug-metabolizing enzymes to aid CCM absorption and bioavailability [229,230]. It has numerous pharmacological effects and some health benefits, particularly against chronic diseases (decrease in insulin resistance, anti-inflammatory properties, amelioration of hepatic steatosis [231]. Shoba et al. (1998) [232] reported that CCM bioavailability improved by 2000% at 45 min after concomitant orally administrated PIP.

Two plausible explanations for the role of PIP in the bioavailability of CCM: (i) nonspecific mechanisms that help to accelerate CCM absorption, such as increasing the blood supply to the gastro-intestinal tract, decreasing the HCl secretion that inhibits the decomposition of various drugs, increasing the emulsifying content of the gut, and increasing the amount of enzymes, such as γ-glutamyl trans-peptidase, which participate in the active and passive transport of nutrients to the intestinal cells, and (ii) nonspecific mechanisms by inhibition of enzymes responsible for the biotransformation of drugs, preventing the inactivation and elimination of CCM [233].

The CCM bioavailability, solubility, and stability, as well as its antioxidant activity were significantly improved by its nano complexation with whey protein concentrate [234,235]. In consequence, the effective role of whey protein concentrate in the cellular penetration and internalization of the CCM-WPC complex and in the specific intracellular trafficking of curcumin including mitochondria or nucleus becomes progressively important as we focus to further enhance the effectiveness of curcumin therapeutic effect.

## 4. Challenges and Perspectives

In the present situation, curcumin holds a strong potential to be used as an anticancer, anti-inflammatory, and chemo-preventive agent with rather good effects on human health. However, the fact that curcumin is poorly absorbed and rapidly metabolized, that only a fraction of the CCM reaches the bloodstream, and that its plasma concentration cannot reach the required therapeutic levels limits its medical applications. In this review, some strategies and numerous studies were given to improve CCM bioavailability. It is worthy to point out that any strategy used to increase the curcumin efficiency will also enhance the associated complications. Thus, CCM delivery systems based on vesicles and nanocarriers, such as proteins and other polymers, can offer a sustained curcumin release in tumor sites, but the reached CCM dosage might not be sufficient for reaching the CCM toxicity.

Future research should focus on the synergistic approaches of curcumin with other bioactive molecules, such as piperine, silymarin, and various structural analogues of CCM, and with anti-cancer drugs. Peroxisome targeted delivery of the CCM to modulate PPAR α/γ signaling in order to decrease the oxidative stress as well as the inflammatory mechanisms via IL-8, VEGF and CD31 expression. miRNA 142 and PSMB5 are new target RNA-protein complexes of curcumin, with large involvements in proteasome enzymes inhibition and this pathway can be a candidate for a newly developed CCM-based therapy in various pathological conditions.

As an overview, a strategy for proteasome inhibition by CCM leads to apoptosis, necrosis, or other cell death mechanisms with important potential usage in antineoplastic therapy, when the cancer cells must be targeted. Instead, CCM bioactivity and bioavailability are codependent, and the dose-related effects are prominent features of CCM in in vivo systems. Low doses of CCM are antioxidants, and high doses of CCM are antitumor factors, however, its anti-inflammatory effects are still understudied related to the dose-dependent effects of curcumin. Moreover, in some cases, inflammation and oxidative status are the critical factors for necrosis with clear advantages in antitumor therapy. On the other side, in light of the CCM’s beneficial effects, the antioxidant, anti-inflammatory, antineoplastic, or other actions are features of an exceptional candidate in natural compounds-based therapy. These aspects need more exploration with various biological models and different doses.

In the foreseen future, the complexation of CCM with multi-proteins from various sources, phospholipids, and fatty acids, using also encapsulation in liposomes, micelles, or with β-cyclodextrin, will jointly develop successful carrier systems for CCM. In addition, these advanced systems might deliver CCM to achieve its augmented serum concentrations to reach curcumin therapeutic levels and simultaneously decrease the elimination of CCM, thus enhancing its bioavailability. A large variety of nanotechnology, based on drug delivery systems, also includes nanoparticles facilitated strategies, including polymeric nanoparticles, such as polyethylene glycol, lipid nanoparticles, and nanogel, which might be used for enhanced CCM delivery and high bioavailability and biological activity of curcumin.

## 5. Conclusions

In the first part of the study, CCM, as a bioactive material, and its disadvantages are presented. The biggest disadvantage of CCM is its low water solubility and thereby low bioavailability; there are many publications on this topic. In this case, CCM contained second- and third-generation solid dispersions, the complexation of CCM with proteins, and the addition of different flavonoids or adjuvants as piperine to CCM were expounded. PEGylation can improve CCM deficiency, but the combination with other techniques or other polymers further improved its properties. Another alternative is the complexation of CCM with proteins, which greatly improves the water dispersibility, stability, and bioavailability of CCM. The best properties of CCM can result from using all the listed techniques together. Recognizing the right preparation process and employing the appropriate excipients in relation to CCM can lead to effective pharmacological treatment by administering a lower dose, which can have an important cost repercussion.

This review provides clues to encourage more research on combined compounds or techniques as a way to improve curcumin’s physical properties, and thus its bioavailability, and its uses in the food and medical areas.

Moreover, current challenges and future perspectives associated with various strategies of curcumin protection, intracellular nanoparticles targeting, and cellular localization may inspire novel studies with the purpose to further improve its therapeutic effect.

## Figures and Tables

**Table 1:** Summary of representative current studies of complexes based on curcumin (CCM), and different types of protein, characterized by primarily fluorescence measurements, F, at pH 7 or 7.4 and different temperatures, and thermodynamic parameters: binding constant, K, and binding number, n, using various types of equations and the changes in the Gibbs free energy, $\Delta G^{o}$ .

Complex of CCM and Various Proteins	Experimental Condition	Equation to Describe the Interaction between CCM and Different Type of Proteins	Thermodynamic Parameters	Ref
Curcumin and β-lactoglobulin CCM-β-Lg complex	Room temperature pH 7.0	$\log\left(\frac{F_{0}-F}{F}\right)={\mathrm{logK}}_{s}+n\log\left[\mathrm{CCM}\right]$ K_S_: binding constant n: binding number [CCM]: curcumin concentration F_0_: fluorescence intensity of protein in the absence of quencher (CCM) F: fluorescence intensity of protein in the presence of quencher	K_S_: 1.33 × 10^5^ M^−1^ n = 1.10 $\Delta G^{o}$: −28.7 kJ/mol	[101]
Curcumin and bovine serum albumin complex CCM-BSA complex	298 K pH 7.4	$\log\left(\frac{F_{0}-F}{F}\right)={\mathrm{nlogK}}_{a}-\mathrm{nlog}\frac{1}{\left(\left[Q_{t}\right]-\frac{\left(F_{0}-F\right)\left[P_{t}\right]}{F_{0}}\right)}$ K_a_: binding constant [Q_t_]: concentration of curcumin [P_t_]: concentration of BSA	K_a_: (0.724 ± 0.01) × 10^5^ L/mol n = 1.110 ± 0.038 $\Delta G^{o}$: −23.05 ± 0.02 kJ/mol	[171]
Curcumin and bovine serum albumin CCM-BSA complex	25 °C pH 7.0	$\log\left(\frac{F_{0}-F}{F}\right)={\mathrm{nlogK}}_{b}-\mathrm{nlog}\frac{1}{\left(\left[Q_{t}\right]-\frac{\left(F_{0}-F\right)}{F_{0}}\left[\mathrm{BSA}\right]\right)}$ K_b_: binding constant	K_b_: 1.95 × 10^5^ L·mol^−1^ n = 1.11 $\Delta G^{o}$: −30.19 kJ·mol^−1^	[172]
Curcumin and soy protein CCM-SP complex	Room temperature pH 12.0	$\frac{F_{0}}{F}=1+K_{\mathrm{SV}}\left[Q\right]$ K_SV_: Stern-Volmer constant [Q]: molar concentration of curcumin	K_SV_: 6.3 × 10^4^ M^−1^ $\Delta G^{o}$: −26.90 kJ⋅mol^−1^	[180]
Curcumin and pea protein isolate CCM-PPI complex	298 K pH 7.0	$\log\left(\frac{F_{0}-F}{F}\right)={\mathrm{logK}}_{a}+\mathrm{nlog}\left[\mathrm{CUR}\right]$ K_a_: binding constant [CUR]: concentration of curcumin	K_a_: 4.74 × 10^4^ L/M n = 0.97 $\Delta G^{o}$: −26.67 kJ·M^−1^	[182]
Curcumin and rice protein CCM-RP complex	298 K pH 12.0	$\frac{F_{0}}{F}=1+K_{\mathrm{SV}}\left[Q\right]$ K_SV_: Stern–Volmer constant [Q]: molar concentration of curcumin	K_SV_: 2.17 × 10^5^ M^−1^ $\Delta G^{o}$: −30.3 kJ/mol	[184]
Curcumin and human serum albumin CCM-HSA complex	298 K pH 7.4	$\log\left(\frac{F_{0}-F}{F}\right)={\mathrm{logK}}_{b}+\mathrm{nlog}\left[Q\right]$ K_b_: binding constant [Q]: quencher concentration n: binding number	K_b_: (1.73 ± 0.32) × 10^7^ M^−1^ $\Delta G^{o}$: −41.29 kJ·mol^−1^	[191]
Curcumin and holo-transferrin CCM-HTR complex			K_b_: (1.81 ± 0.32) × 10^6^ M^−1^ $\Delta G^{o}$: −36.46 kJ·mol^−1^	
Curcumin and human serum albumin and holo-transferrin CCM-HSA-HTR complex			K_b_: (3.19 ± 0.32) × 10^6^ M^−1^ $\Delta G^{o}$: −37.98 kJ·mol^−1^	
Curcumin and whey protein isolate CCM-WPI complex	308 K pH 7.0	$\log\left(\frac{F_{0}-F}{F}\right)={\mathrm{logK}}_{a}+\mathrm{nlog}\left[Q\right]$ K_a_: binding constant	K_a_: 1.08 × 10^6^ L·mol^−1^ n = 1.30 $\Delta G^{o}$: −37.08 kJ·mol^−1^	[192]
Curcumin and pea protein isolate CCM-PPI complex			K_a_: 7.68 × 10^6^ L·mol^−1^ n = 1.44 $\Delta G^{o}$: −41.00 kJ·mol^−1^	
Curcumin and β-casein CCM-β-casein complex	298 K pH 7.4	$\log\left(\frac{F_{0}-F}{F}\right)={\mathrm{logK}}_{b}+\mathrm{nlog}\left[Q\right]$ K_b_: binding constant	K_b_: (3.98 ± 0.26) × 10^4^ L·mol^−1^ n = 1.59 ± 0.14 $\Delta G^{o}$: −26.2 ± 0.09 kJ·mol^−1^	[193]
Curcumin and β-lactoglobulin CCM-β-Lg complex	25 °C pH 7.4	$\frac{F_{0}-F}{F_{0}-F_{C}}=\frac{{\left[P\right]}_{t}+{\left[Q\right]}_{a}+K_{d}-\sqrt{{\left({\left[P\right]}_{t}+{\left[Q\right]}_{a}+K_{d}\right)}^{2}-4{\left[P\right]}_{t}{\left[Q\right]}_{a}\;}}{2\times {\left[P\right]}_{t}}$ K_d_: dissociation constant; the reciprocal of K_d_ is K_a_ K_a_: binding constant; [Q]_a_ is the concentration of added CCM $F_{C}$: fluorescence of the fully complexed protein	K_a_: 1.19 × 10^4^ M^−1^ $\Delta G^{o}$: −23.2 kJ·mol^−1^	[196]
Curcumin and bovine lactoferrin CCM-β-Lacto complex	299.15 K pH 7.4	$\log\left(\frac{F_{0}-F}{F}\right)={\mathrm{logK}}_{b}+\mathrm{nlog}\left[Q_{t}\right]$ K_b_: binding constant	K_b_: (3.01 ± 0.16) × 10^4^ M^−1^ n = 1.03 ± 0.16 $\Delta G^{o}$: −25.65 kJ·mol^−1^	[198]

## Data Availability

Not applicable. Figure 4 and Figure 5 were created using the BioRender software/28–29 September 2022.
